# Differences in the Asymmetry of Beat-to-Beat Fetal Heart Rate Accelerations and Decelerations at Preterm and Term Active Labor

**DOI:** 10.3390/s21248249

**Published:** 2021-12-10

**Authors:** Carolina López-Justo, Adriana Cristina Pliego-Carrillo, Claudia Ivette Ledesma-Ramírez, Hugo Mendieta-Zerón, Miguel Ángel Peña-Castillo, Juan Carlos Echeverría, Jorge Rodríguez-Arce, José Javier Reyes-Lagos

**Affiliations:** 1 Facultad de Medicina (School of Medicine), Universidad Autónoma del Estado de México (Autonomous University of Mexico State), Toluca de Lerdo 50180, Mexico; clopezj234@alumno.uaemex.mx (C.L.-J.); acpliegoc@uaemex.mx (A.C.P.-C.); ciledesmar@uaemex.mx (C.I.L.-R.); hmendietaz@uaemex.mx (H.M.-Z.); 2 Hospital Materno Perinatal Mónica Pretelini Sáenz, Instituto de Salud del Estado de México (Health Institute of Mexico State), Toluca de Lerdo 50010, Mexico; 3 División de Ciencias Básicas e Ingeniería (Basic Science and Engineering Division), Universidad Autónoma Metropolitana Unidad Iztapalapa (Metropolitan Autonomous University Campus Iztapalapa), Iztapalapa 09340, Mexico; mapc@xanum.uam.mx (M.Á.P.-C.); jcea@xanum.uam.mx (J.C.E.); 4 Facultad de Ingeniería (School of Engineering), Universidad Autónoma del Estado de México (Autonomous University of Mexico State), Toluca de Lerdo 50100, Mexico; jrodrigueza@uaemex.mx

**Keywords:** phase-rectified signal averaging, fetal heart rate, multiscale asymmetry, moderate premature labor

## Abstract

The fetal autonomic nervous system responds to uterine contractions during active labor as identified by changes in the accelerations and decelerations of fetal heart rate (FHR). Thus, this exploratory study aimed to characterize the asymmetry differences of beat-to-beat FHR accelerations and decelerations in preterm and term fetuses during active labor. In an observational study, we analyzed 10 min of fetal R-R series collected from women during active preterm labor (32–36 weeks of pregnancy, n = 17) and active term labor (38–40 weeks of pregnancy, n = 27). These data were used to calculate the Deceleration Reserve (*DR*), which is a novel parameter that quantifies the asymmetry of the average acceleration and deceleration capacity of the heart. In addition, relevant multiscale asymmetric indices of FHR were also computed. Lower values of *DR*, calculated with the input parameters of *T* = 50 and *s* = 10, were associated with labor occurring at the preterm condition (*p* = 0.0131). Multiscale asymmetry indices also confirmed significant (*p* < 0.05) differences in the asymmetry of FHR. Fetuses during moderate premature labor may experience more decaying R-R trends and a lower magnitude of decelerations compared to term fetuses. These differences of FHR dynamics might be related to the immaturity of the fetal cardiac autonomic nervous system as identified by this system response to the intense uterine activity at active labor.

## 1. Introduction

The fetal heart rate (FHR) shows specific patterns during labor owing to the rhythmic contractions in the woman’s uterus. The regular contractions generate repetitive decelerations and subsequent accelerations of the FHR [1,2,3]. These patterns are associated with the response of the fetal parasympathetic and sympathetic autonomic nervous system (ANS) to the transitory reduction of the oxygen bloodstream contents required in vital organs to conserve life and avoid hypoxic injury [4].

Preterm labor occurs before 37 weeks of gestation [5], and it is considered as the most important risk cause for infant death below five years [6]. The authors have incorporated the measurement of cardiac fetal autonomic activity during labor as a valuable tool to assess fetal well-being [2]. Hence, continuous and accurate fetal monitoring could be considered to guarantee mother and child’s health during labor. However, in the current clinical practice, there is a high degree of subjectivity or a lack of medical dexterity to identify FHR accelerations and decelerations by visual evaluation [2,7,8]

The phase rectified signal averaging (*PRSA*) approach represents an indirect integrated quantification of the ANS; this approach is used to identify subtle short-term repeated patterns (i.e., quasiperiodicities) in a time signal that are normally masked by nonstationarities [9]. The main advantage of *PRSA* is its high responsiveness to non-stationary signals such as the FHR [10]. The average deceleration capacity (*ADC*) and average acceleration capacity (*AAC*) are indices obtained by the *PRSA* algorithm that show high sensitivity to discriminate among various clinical and preclinical conditions using FHR traces, such as fetal distress [11], cardiovascular risk [10], and intrauterine growth restriction [12,13]. These parameters are used to assess the heart rate “capacity” to either decrease or increase; thus, *AAC* and *ADC* have been related to the fetal sympathetic and parasympathetic activity, respectively [9].

Given that *AAC* and *ADC* show similar values in identical experimental tests [14,15], these parameters may appear limited though to recognize changes in the autonomic regulation. Therefore, a new parameter, the Deceleration Reserve (*DR*), has been introduced [16]. The *PRSA* algorithm is also used to compute *DR*, and it is related to the sum between *AAC* and *ADC*. This parameter emphasizes the asymmetric trends of heart rate accelerations and decelerations that occur as a consequence of stress situations that involve ANS changes, and it has the advantage that can be applied to analyze non-stationary signals [3,16].

The *DR* index was previously studied by Rivolta et al. in a fetal hypoxia sheep model, concluding that *DR* showed better discrimination capacity than *AAC* and *ADC* [16]. In addition, this study reported lower *DR* values for chronically more hypoxic than normoxic fetuses; the authors concluded that *DR* is a potential parameter of fetal well-being during pregnancy. Similarly, the study demonstrated *DR*’s potential to discriminate between fetuses diagnosed with acidemia at birth and normal fetuses [16]. 

Emerging evidence has even suggested that *DR* may be considered to measure the fetal heart rate’s asymmetry, providing more precise monitoring of the ANS dynamics even when there is a lack of data and noise [16,17]. Asymmetry is a fundamental characteristic of non-equilibrium systems [18]; hence, this property is expected to be present and has been documented in physiological systems [19]. Furthermore, asymmetry is linked to the system’s time-irreversibility, which is also manifested in a healthy physiologic system [20]. For example, heart rate dynamics show complex multiscale irreversibility in healthy subjects, such that not only the originals but even coarse-grained time series are asymmetric across a wide variety of scales [20,21]. In the fetal context, other studies indicate that the asymmetry of fetal heart rate fluctuations changes along fetal development, and these changes correlate with the sympathetic activity progressing toward delivery [22].

Considering this background, our study here aimed to compare the heart rate dynamics in preterm and term fetuses during active labor by (a) computing *DR* using different input values of the *PRSA* algorithm and (b) assessing asymmetry by applying other relevant multiscale indices. Given that *DR* has proven to be a sensitive parameter to detect diverse fetal hypoxic conditions, we hypothesized that preterm fetuses exhibit lower *DR* values of fetal heart rate and more symmetric behavior than term fetuses during active labor. The value of this exploratory research lies in that it offers theoretical contributions to understand the asymmetric dynamics of beat-to-beat fetal heart rate during term and preterm labor. Thus, changes in the asymmetry of fetal heart rhythm during labor could be used eventually to distinguish differences in the autonomic regulation, paving the way for new monitoring strategies of the fetal condition.

## 2. Materials and Methods

### 2.1. Data Collection

In this study, we recorded transabdominal data of pregnant women during active labor at term (38–40 weeks of gestation by pelvic ultrasound, Group Term) and women with a medical diagnosis of moderate preterm labor (32–36 weeks of gestation by pelvic ultrasound, Group Preterm) who attended the “Mónica Pretelini Sáenz” Maternal-Perinatal Hospital, Toluca, State of Mexico, Mexico. The ethics committee from this institution approved this study (reference number: 2018-10-607). Written informed consent was obtained from each participant, and the study was conducted according to the ethical standards of the Declaration of Helsinki and following the relevant guidelines and regulations. 

Active labor was identified by the manifestation of at least four contractions in 10 min, 50% cervical effacement, and 4 cm of dilation. Women with twin pregnancy, gestational diabetes, hypertensive disorders, epidural block during labor, and chronic degenerative diseases were excluded for both groups.

A total of 87 maternal/fetal dyads during preterm and active labor were enrolled in this study. However, 43 studies were excluded from the analysis for one or more of the following reasons: high signal losses of FHR data; lack of continuous 10 min of FHR without outliers or data missing; incomplete clinical information; and poor signal quality. Thus, only seventeen recordings conformed to the Preterm group (n = 17) and twenty-seven conformed to the Term group (n = 27).

We utilized a fetal-maternal device (Monica AN24, Monica Healthcare, Nottingham, UK). The MonicaAN24 device is a validated system to detect precise fetal cardiac time intervals from 32 weeks’ gestational age onwards [23,24]. According to Reinhard et al., intrapartum FHR monitoring via the abdominal electrocardiogram (ECG) offers diminished ‘ambiguous fetal heart rate’ traces when compared to cardiotocography [25]. The bioelectric data were recorded for 30 min using disposable electrodes (Ambu BlueSensor VL, Ambu A/S, Ballerup, Denmark) in a bipolar configuration. After cleaning the abdominal surface with an alcohol swab, the sensors were placed after gently abrading the skin with an abrasive material to reduce skin impedance. The sampling frequency of the recordings was 900 Hz.

### 2.2. Segmentation of Fetal RR Time Series and Preprocessing

The fetal beat-to-beat R-R intervals were obtained using the Monica DK software (Monica Healthcare, UK). To avoid the artifacts caused by fetal or maternal movements that could alter the measurement of fetal R-R intervals, we visually selected a continuous 10 min of fetal R-R intervals without outliers in the presence of three or four uterine contractions. Adaptive filtering was also used to eliminate any ectopic beats from the segmented time series, consisting of three steps: eliminating obvious recognition errors, percent adaptive filtering, and adaptive control filtering [26]. The quality of fetal beat-to-beat R-R intervals was addressed as the percentage of missed heartbeats less than 10% in both groups.

### 2.3. Definition of AAC and ADC

The average deceleration capacity and average acceleration capacity (*ADC* and *AAC*, respectively) were calculated by the Phase-Rectified Signal Averaging (*PRSA*) algorithm proposed by Bauer et al. [27]. The *AAC*, *ADC*, and *DR* values strongly depend on the input values *T*, *L*, and *s* of the *PRSA* algorithm. Specifically, *T* establishes the number of points of the low-pass moving average filter to identify the anchor points. *L* defines the *PRSA* signals length, and it is required to be longer than the period of the slowest fluctuation to be identified with *PRSA*. The parameter *s* determines the fluctuations in the *PRSA* signals that most impact *AAC* and *ADC* values [14]. Interestingly, a range of values has been reported for which the *PRSA* algorithm seems to provide important physiological information of the fetal condition [13].

The first step of the *PRSA* algorithm is the definition of anchor points of the R-R series, which are given by:

For deceleration anchors points:

(1) $$\frac{1}{T}\sum_{t=0}^{T-1}fRR\left(t+1\right)>\frac{1}{T}\sum_{t=1}^{T}fRR\left(t-1\right)$$

These points correspond to decrements in the time series. To calculate the acceleration anchor points, the sign of the inequality of Equation (1) is reversed. 

Subsequently, concerning each of the anchor points, windows of length *L* are defined. These windows are aligned to be averaged, taking as reference such anchor points. The resulting signal of length 2*L* + 1 is the *PRSA* signal.

The *PRSA* signal is used to calculate the *ADC* and *AAC* parameters, as follows: [28]

(2) $$ADC=\frac{1}{2s}\sum_{i=1}^{s}PRSA\left(L+i\right)-\frac{1}{2s}\sum_{i=0}^{s-1}PRSA\left(L-i\right)$$

*AAC* is calculated with the equivalent Formula (2) but with the corresponding *PRSA* signal obtained by employing instead the accelerations anchor points. According to relevant evidence, the *AAC* and *ADC* indices applied to fetal R-R time series describe the speed of changes in fetal heart rate, triggered by sympathetic and vagal branches, reflecting fetal ANS [28].

### 2.4. Deceleration Reserve (DR)

Deceleration Reserve (*DR*) is a new parameter introduced by Rivolta et al.; it is given by the sum of *ADC* and *AAC*:

(3) DR=ADC+AAC

**Figure 1:**
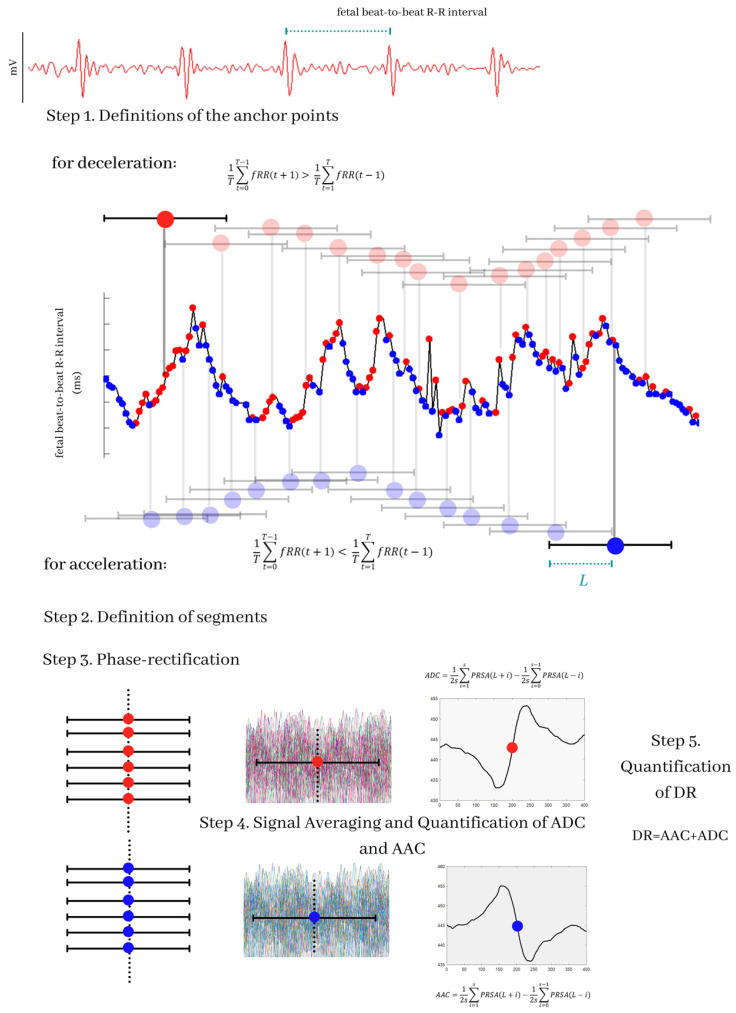
Description of the Phase-Rectified Signal Averaging (*PRSA*) algorithm and of the generation of the *ADC*, *AAC*, and *DR* indices. The transabdominal ECG signals are processed to obtain the fetal beat-to-beat R-R time series. The first step of the *PRSA* algorithm is the selection of the anchor points; in the figure, the anchor points for decelerations are marked with red points, and the anchor points for accelerations are marked with blue points; the selection of the anchor points depends on the parameter *T*. The second part of the algorithm is the choice of a time window covering each anchor point; this window measures 2*L*. The next step of the algorithm is a phase-rectification; then, all the time windows are aligned and averaged with respect to the anchor points. This process provides the *PRSA* signal. The *ADC* and *AAC* values are the central point of the signal. Finally, *DR* values are calculated by the sum of *AAC* and *ADC*.

Similar to *AAC* and *ADC*, *DR* depends on the input parameters *L*, *T*, and *s,* which are taken from the *PRSA* algorithm (Figure 1). *DR* indicates whether the average increase in the time series is principally formed by increasing (*DR* is positive) or decreasing trends (*DR* is negative). For this study, we calculated *DR* considering *T* in a range of 1 to 50 and *s* in a range of 1 to 10 (for each value of *T)* using a fixed value *L* = 50, which were the range and values proposed as appropriate in a previous study [16].

### 2.5. Multiscale Asymmetry Indices

The motivation to introduce the multiscale asymmetry indices in this research comes from the fact that *DR* was inspired by studies about the effects of heart rate asymmetry on *AAC* and *ADC* performed by Pan et al. [29] and Karmakar et al. [22].

By considering the time series constructed by the differences signal ΔRR[n]=RR[n+1]−RR[n], 0 ≤ *n* ≤ *L* − 2 (in which *L* is the length of the fetal beat-to-beat R-R intervals time series), we can define $\Delta RR^{+}$ and $\Delta RR^{-}$ as a signal with only the positive and negative values of ΔRR, respectively. Thus, the following indices suitable for short-term heart rate recordings were used to quantify the asymmetry of fetal beat-to-beat R-R intervals using the Pybios software [30]. 

Porta’s index (*PI*%) is based on calculating the percentage of negative $\Delta RR^{-}$ with respect to the total number of ΔRR≠0. This index can be computed as:

(4) $$PI\%=\frac{N\left(\Delta RR^{-}\right)}{N\left(\Delta RR\neq 0\right)}\cdot 100$$

*PI*% values greater than 50 indicate that the number of negative $\Delta RR^{-}$ (i.e., accelerations) are larger than the number of positive $\Delta RR^{+}$ (i.e., decelerations) [31]. 

The Guzik index (*GI*%) is based on the assessment of the percentage of the sum of the square values of positive $\Delta RR^{+}$ to the cumulative square values of all ΔRR. It is expressed as:

(5) $$GI\%=\frac{\sum_{i=1}^{N\left(\Delta RR^{+}\right)}\Delta RR^{+}{\left(i\right)}^{2}}{\sum_{i=1}^{N\left(\Delta RR\right)}\Delta RR{\left(i\right)}^{2}}\cdot 100$$

*GI*% is an index that allows the computation of the decelerations/accelerations influence into short-term heart rate data. Different from *PI*%, the computation of *GI*% considers the weight of the positive differences between two *RR* intervals. 

Ehlers’ index (*EI*) is based on assessing the skewness of the distribution of Δ*RR*. It is defined as:

(6) $$EI=\frac{\sum_{i=1}^{N\left(\Delta RR\right)}\Delta RR{\left(i\right)}^{3}}{{\left(\sum_{i=1}^{N\left(\Delta RR\right)}\Delta RR{\left(i\right)}^{2}\right)}^{3/2}}$$

*EI* values far from 0 indicate that the series is asymmetric. If *EI* > 0, the distribution of ΔRR is skewed toward positive values and, thus, the averaged magnitude of $\left|\Delta RR^{+}\right|$ is larger than that of $\left|\Delta RR^{-}\right|$. *PI*% and *G*% values that are significantly lower than 50 and *EI* values that are significantly lower than 0 indicate that ΔRR is skewed toward negative values [31].

The multiscale asymmetry method corresponds to the calculation of *PI*%, *GI*%, and *EI* for numerous τ—scaled versions of the original R-R time series. For a given time series ΔRR of length *L*, each point in a τ—scaled signal is defined as $\Delta RR^{\tau }\left[n\right]=\frac{1}{\tau }\overset{n\tau }{\underset{i=\left(n-1\right)\tau +1}{\sum }}RR\left[i\right],\;1\leq n\leq L/\tau$. Then, *PI*%, *GI*%, and *EI* are calculated for all $\Delta RR^{\tau }\left[n\right]$ signals. In this study, we used a scale (τ = 1 to 10) to analyze fetal R-R intervals’ asymmetry of accelerations and decelerations.

### 2.6. Statistical Analysis

Normal distribution was tested by the D’Agostino and Pearson test for all indices calculated from the Term and Preterm groups. A one-tailed, unpaired t-test was used to compare the mean values of the *PRSA* indices *AAC*, *ADC*, and *DR* as well as the multiscale asymmetry ones (*PI*%, *GI*%, and *EI*) if normality was accepted. Otherwise, as a nonparametric alternative, the Mann–Whitney test was applied. For all tests, results of *p* < 0.05 were considered significant. 

In this study, each statistical analysis was carried out using the GraphPad Prism version 8.00 for Windows (GraphPad Software, La Jolla, CA, USA).

## 3. Results

### 3.1. Clinical Characteristics

Maternal and infant clinical characteristics of the Term (n = 27) and Preterm (n = 17) groups are shown in Table 1. We confirmed differences (*p* < 0.05) between Term and Preterm groups in expected clinical characteristics such as gestational age: 39 ± 1 vs. 34 ± 2 weeks, newborn birthweight: 2.9 ± 0.4 kg vs. 2.4 ± 0.6 kg, head circumference: 33.7 ± 1.73 cm vs. 32.0 ± 2.29 cm, and fetal size: 49.5 ± 2.1 cm vs. 45.1 ± 6.0 cm, respectively. Differences in these newborns’ clinical characteristics between the Term and Preterm groups confirmed the medical diagnosis of moderate prematurity. No maternal differences concerning age, body mass index, cervical dilatation, and effacement between groups were presented.

### 3.2. PRSA

None of the mean values of *AAC* or *ADC* indices exhibited differences (*p* > 0.05) between the Term and Preterm groups at any of the values of *T* and *s* evaluated (data not shown). However, *DR* did reveal significant differences between Term and Preterm for various values of *T* and *s*. With *T* = 50 and *s* = 10, the lowest *p*-value (*p* = 0.0131) was achieved followed by *T* = 50 and *s* = 1 (*p* = 0.0147, Table 2). Interestingly, the median *DR* values were lower (negative, decreasing trends) in the Preterm compared to Term (positive, increasing trends) for most cases.

**Figure 2:**
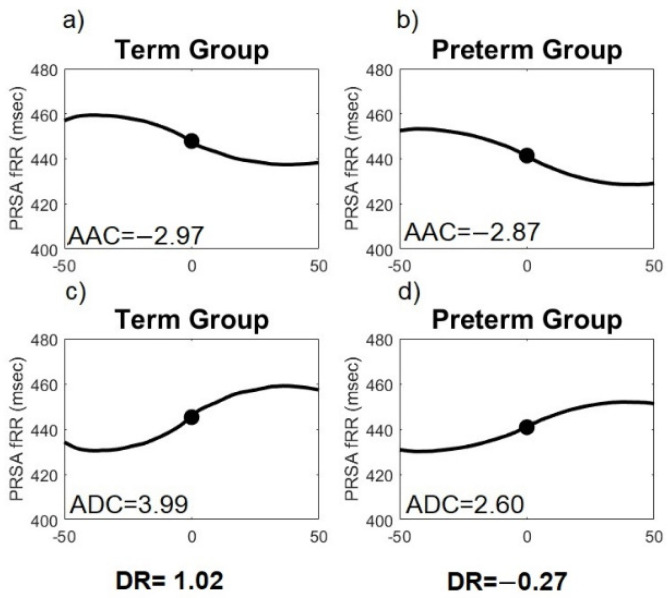
Examples of Phase-Rectified Signal Averaging (*PRSA*) curves (*T* = 50, *s* = 10) of fetal heart rate for the average acceleration capacity (*AAC*) and average deceleration capacity (*ADC*) in Preterm and Term active labor. (**a**) *PRSA-AAC* curve for Term; (**b**) *PRSA-AAC* curve for Preterm; (**c**) *PRSA-ADC* curve for Term and (**d**) *PRSA-ADC* curve for Preterm. A reduction of the central part of the *ADC* and *AAC* curve in Preterm can be observed.

Representative *PRSA* curves for *T* = 50 and *s* = 10 obtained from the fetal beat-to-beat R-R intervals time series in Preterm and Term fetuses are exhibited in Figure 2. Quantitative analysis of *AAC* and *ADC* is based on the assessment of the central section of the *PRSA* curve. The unit of measurement of *ADC* and *AAC* is reported in milliseconds. 

### 3.3. Multiscale Asymmetry Indices

**Figure 3:**
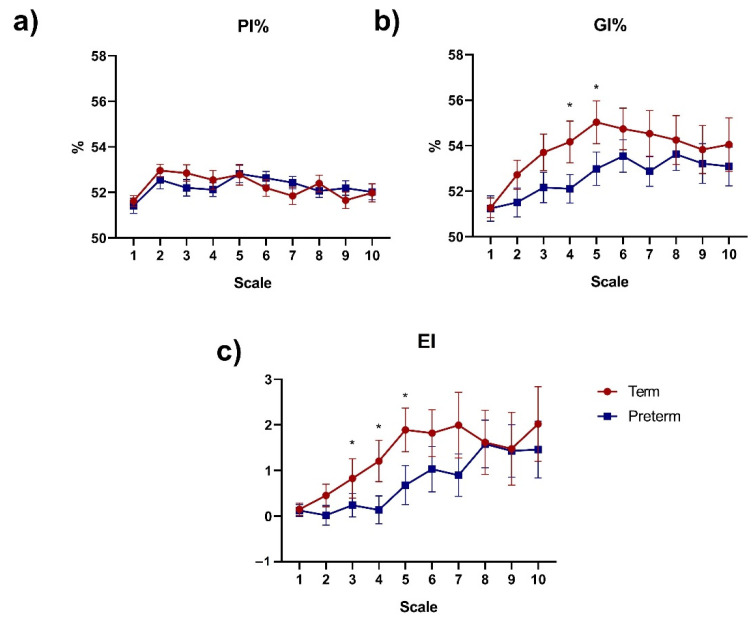
Error bar (mean ± SEM) of multiscale asymmetry indices of fetal beat-to-beat R-R intervals time series using (**a**) Porta (PI%); (**b**) Guzik (GI%); and (**c**) Ehlers (EI) at lags τ = 1–10 for Preterm and Term groups. * *p* < 0.05 between Term and Preterm (unpaired t-test or Mann–Whitney test).

Figure 3 shows the results obtained for the PI%, GI%, and EI indices at different scales between the Term and Preterm groups. PI% did not exhibit significant differences for any of the scales (Figure 3a). Additionally, GI% (Figure 3b) showed significant differences (*p* < 0.05) between Term and Preterm at scales 4 (54% ± 5 vs. 52% ± 3, *p* = 0.03) and 5 (55% ± 5 vs. 53% ± 3, *p* = 0.04), respectively. GI% mean values of the Term group were larger for the scales 1 to 5 and smaller for subsequent scales. This behavior was not observed in the Preterm group. Noteworthy, the mean values of GI% for the Term group were higher in all scales evaluated compared to the Preterm group. Moreover, EI mean values (Figure 3c) presented significant differences between Term and Preterm groups at scales 3 (0.82 ± 2.23 vs. 0.24 ± 1.06, *p* = 0.02), 4 (1.20 ± 2.34 vs. 0.13 ± 1.25, *p* = 0.03) and 5 (1.89 ± 2.48 vs. 0.68 ± 1.75, *p* = 0.03). 

## 4. Discussion

According to the consulted literature, this is the first study that assesses the asymmetry of beat-to-beat FHR by applying *DR* in a wide range of *T* and *s* values in comparison with the calculation of other multiscale asymmetry indices for fetuses during active term and preterm labor. Our results indicate that *DR* obtained from the *PRSA* algorithm is better than *AAC*, *ADC*, and multiscale asymmetry indices to identify differences of FHR between labor preterm and term conditions. Particularly, the most appropriate input values of *PRSA* to discriminate between preterm and term fetuses using *DR* were *T* = 40, *s* [1–4]; *T* = *45*, *s* [1–10]; *T* = *50*, *s* [1–10]. Notably, *T* = 50, *s* = 10 showed the lowest *p*-value (*p* = 0.0131) (Table 2) in the comparison between those groups. Although optimal *T* and *s* values have been found under certain preclinical conditions [13], their physiological significance is still unclear. Thus, these “optimal values” may change according to the signals or time series analyzed with the *PRSA* algorithm.

A high *T* value (*T* = 50) is associated with low frequencies of the fetal beat-to-beat R-R intervals in the *PRSA* signal calculation. Since the low frequencies of the fetal beat-to-beat R-R intervals time series could be related to the sympathetic and parasympathetic systems’ response, as noted in [1,32,33,34], we speculate that both autonomic branches manifest different activity during labor between the term and preterm fetuses. Interestingly, we found that several mean values of *DR* were negative in the Preterm group (decreasing trends), while positive values were found mainly for the Term group (increasing trends). A positive or negative *DR* value reflects the fact that the average behavior in the FHR time series is mainly composed of growing or decaying heart rate trends, respectively [16].

According to our previous results, during parturition, the short-term fetal heart rate variability is decreased, showing decreased vagal modulations and higher adrenergic response of the heart [35]. Consequently, fetuses during moderate premature labor may experience late or restricted physiological compensatory responses that could be related to the immaturity of the autonomic nervous system [36]. The comparing results obtained by the multiscale asymmetry indices confirmed asymmetric differences in FHR during the preterm condition. Interestingly, relevant evidence indicates that FHR asymmetry increases after 35 weeks’ gestation compared to before 32 weeks in nonlaboring women [22].

According to relevant evidence [37], GI% was decreased (lower asymmetric) during a stress protocol in newborns, suggesting that heart rate asymmetry may be considered a new marker for neonatal stress. It has been documented that some time series (e.g., healthy heart rate dynamics) are asymmetric over a wide range of scales [38]. Additionally, GI% is also associated with the magnitude of decelerations present in the R-R signal [22]. Our results show that GI% and EI values were increased in Term compared to Preterm, suggesting larger decelerations in the Term condition during labor. As previous evidence has revealed [39], an increment of GI% is associated with increased parasympathetic nervous system activity. Thus, we hypothesize that fetuses during moderate premature labor might exhibit a different response to stress during active labor than term fetuses. The findings presented here are in line with those of Hurtado-Sánchez et al., who reported that preterm fetuses manifested slightly higher FHR and lower amplitude decelerations compared to term fetuses evaluated by cardiotocography [40].

The parasympathetic influence of any ongoing beat increases with a larger lag (distant heartbeats) in the Term group. The results of a previous study indicate that GI% and EI performed better than PI% in detecting changes in the asymmetry in cardiovascular signals in psychopathological scenarios [41].

This work has the following limitations. Our final groups of participants that provided bioelectrical signals at Preterm (n = 17) and Term active labor (n = 27) were small due to the inherent difficulty of successfully collecting continuous 10 min data of fetal beat-to-beat RR time series. However, even with a small sample, we were able to find statistically significant differences between preterm and term conditions using *DR* values. Other studies have also detected significant changes in *PRSA* parameters using a small sample population [42]. *DR* was recently introduced in perinatal research, and its application has been studied in few preclinical and clinical datasets [16]. Additionally, the *L* value was fixed to 50 in all the evaluations of the *PRSA* algorithm. Future studies are needed to evaluate the effect of diverse values of *L* in the *DR* calculation. Nevertheless, some studies have reported that the *L* value is not as critical for a proper *PRSA* computation [43]. Finally, forthcoming research of computerized intrapartum monitoring should be directed toward investigating multiple parameters derived from fetal heart rate and the application of novel machine-learning techniques for data analysis [44]. A more comfortable recording experience by using a single abdominal sensor is also suggested [45] in conjunction with novel measurements of FHR [46].

## 5. Conclusions

Fetuses in moderate preterm active labor may experience more decaying R-R trends and a lower magnitude of decelerations of heart rate compared to term fetuses. These asymmetric differences of fetal heart rate dynamics might be related to the immaturity of the fetal cardiac autonomic nervous system as identified by this system response to the intense uterine activity at active labor. Thus, the asymmetry of accelerations and deceleration of beat-to-beat FHR is a promising complementary parameter in monitoring fetal well-being during labor. Our findings suggest that the *DR* with *T* = 50 and *s* = 10 demonstrated to be a superior discrimination tool than *AAC*, *ADC*, and multiscale asymmetry indices to identify physiological FHR differences between preterm and term labor.

## Figures and Tables

**Table 1:** Clinical characteristics of the mother and newborn.

	Term	Preterm
	(n = 27)	(n = 17)
Maternal age (years)	21 ± 4	21 ± 4
Weeks of gestation (weeks, USG)	^a^ 39 ± 1	34 ± 2
Maternal BMI (kg/cm^2^)	24.3 ± 1.3	25 ± 2.8
Cervical dilatation (cm)	5.9 ± 1.6	5.0 ± 1.7
Cervical effacement (%)	71 ± 12	62 ± 13
Newborn birth weight (kg)	^a^ 2.9 ± 0.4	2.4 ± 0.6
APGAR score 1 min (>7)	96%	80%
APGAR score 5 min (>7)	96%	70%
Head circumference (cm)	^a^ 33.7 ± 1.73	32.0 ± 2.29
Fetal size (cm)	^a^ 49.5 ± 2.1	45.1 ± 6.0
Gender (male percentage)	52%	50%
R-R mean (ms)	^a^ 431.2 ± 31.0	413.2 ± 26.9

^a^*p* < 0.05 between Term and Preterm (Mann–Whitney test).

**Table 2:** Significant *DR* values (median and interquartile range in (ms) for the Term and Preterm groups.

*T*	*s*	Term	Preterm	*p*-Value
		n = 27	n = 17	
40	1	0.02 (−0.00, 0.05)	0.01 (−0.02, 0.04)	0.0470
40	2	0.04 (−0.00, 0.09)	0.00 (−0.03, 0.07)	0.0487
40	3	0.07 (0.00, 0.14)	0.00 (−0.05, 0.11)	0.0483
40	4	0.09 (0.00, 0.19)	0.01 (−0.07, 0.14)	0.0496
45	1	0.02 (−0.01, 0.04)	−0.00 (−0.02, 0.03)	0.0296
45	2	0.03 (−0.01, 0.09)	−0.01 (−0.04, 0.06)	0.0277
45	3	0.05 (−0.02, 0.13)	−0.01 (−0.06, 0.08)	0.0294
45	4	0.07 (−0.02, 0.02)	−0.02 (−0.08, 0.11)	0.0324
45	5	0.08 (−0.03, 0.21)	−0.02 (−0.10, 0.13)	0.0342
45	6	0.09 (−0.04, 0.25)	−0.02 (−0.12, 0.15)	0.0359
45	7	0.11 (−0.05, 0.28)	−0.03 (−0.14, 0.18)	0.0380
45	8	0.13 (−0.05, 0.31)	−0.03 (−0.16, 0.20)	0.0397
45	9	0.14 (−0.06, 0.34)	−0.04 (−0.17, 0.22)	0.0413
45	10	0.17 (−0.04, 0.48)	−0.04 (−0.19, 0.23)	0.0191
50	1	0.02 (−0.01, 0.05)	−0.00 (−0.02, 0.02)	0.0147
50	2	0.03 (−0.02, 0.10)	−0.01 (−0.05, 0.05)	0.0162
50	3	0.04 (−0.03, 014)	−0.01 (−0.09, 0.08)	0.0186
50	4	0.06 (−0.04, 0.19)	−0.02 (−0.11, 0.10)	0.0208
50	5	0.07 (−0.04, 0.23)	−0.02 (−0.13, 0.12)	0.0223
50	6	0.07 (−0.04, 0.28)	−0.03 (−0.15, 0.14)	0.0236
50	7	0.06 (−0.05, 0.31)	−0.02 (−0.17, 0.16)	0.0252
50	8	0.06 (−0.06, 0.35)	−0.03 (−0.19, 0.18)	0.0266
50	9	0.06 (−0.06, 0.38)	−0.03 (−0.21, 0.21)	0.0280
50	10	0.09 (−0.05, 0.49)	−0.03 (−0.23, 0.22)	0.0131
